# Clinical markers of asthma and IgE assessed in parents before conception predict asthma and hayfever in the offspring

**DOI:** 10.1111/cea.12906

**Published:** 2017-03-28

**Authors:** R. J. Bertelsen, M. Rava, A. E. Carsin, S. Accordini, B. Benediktsdóttir, J. Dratva, K. A. Franklin, J. Heinrich, M. Holm, C. Janson, A. Johannessen, D. L. Jarvis, R. Jogi, B. Leynaert, D. Norback, E. R. Omenaas, C. Raherison, J. L. Sánchez‐Ramos, V. Schlünssen, T. Sigsgaard, S. C. Dharmage, C. Svanes

**Affiliations:** ^1^ Department of Clinical ScienceUniversity of BergenBergenNorway; ^2^ Department of Occupational MedicineHaukeland University HospitalBergenNorway; ^3^ INSERM U1168, VIMA: Aging and Chronic DiseasesEpidemiological and Public Health ApproachesVillejuifFrance; ^4^ UMR‐S 1168Univ Versailles St‐Quentin‐en‐YvelinesMontigny le BretonneuxFrance; ^5^ Genetic and Molecular Epidemiology GroupSpanish National Cancer Research Center (CNIO)MadridSpain; ^6^ ISGlobalCentre for Research in Environmental Epidemiology (CREAL)BarcelonaSpain; ^7^ Universitat Pompeu FabraBarcelonaSpain; ^8^ CIBER de Epidemiología y Salud Pública (CIBERESP)BarcelonaSpain; ^9^ Unit of Epidemiology and Medical StatisticsDepartment of Diagnostics and Public HealthUniversity of VeronaVeronaItaly; ^10^ Medical FacultyUniversity of IcelandReykjavíkIceland; ^11^ Department of Epidemiology and Public HealthSwiss Tropical and Public Health InstituteBaselSwitzerland; ^12^ Department of Surgical and Perioperative SciencesUmeå UniversityUmeåSweden; ^13^ Helmholtz Zentrum MünchenGerman Research Center for Environmental HealthInstitute of Epidemiology INeuherbergGermany; ^14^ Institute and Outpatient Clinic for Occupational, Social, and Environmental MedicineLudwig Maximilians University MunichMunchenGermany; ^15^ Department of Occupational and Environmental MedicineSahlgrenska University HospitalGothenburgSweden; ^16^ Department of Medical SciencesUppsala UniversityUppsalaSweden; ^17^ Centre for International HealthDepartment of Global Public Health and Primary CareUniversity of BergenBergenNorway; ^18^ Centre for Clinical ResearchHaukeland University HospitalBergenNorway; ^19^ Respiratory Epidemiology, Occupational Medicine and Public HealthNational Heart and Lung InstituteImperial CollegeLondonUK; ^20^ Tartu University HospitalLung ClinicTartuEstonia; ^21^ Inserm, UMR 1152Pathophysiology and Epidemiology of Respiratory Diseases, Epidemiology TeamParisFrance; ^22^ UMR 1152University Paris Diderot Paris 7ParisFrance; ^23^ INSERM U897 Bordeaux UniversityBordeaux CedexFrance; ^24^ Department of NursingUniversity of HuelvaHuelvaSpain; ^25^ Department of Public HealthAarhus UniversityAarhusDenmark; ^26^ National Research Centre for the Working EnvironmentCopenhagenDenmark; ^27^ Allergy and Lung Health Unit, Melbourne School of Population HealthThe University of MelbourneMelbourneVic.Australia

**Keywords:** asthma, clinical immunology, European Community Respiratory Health Survey, epidemiology, IgE, offspring, preconception, rhinitis

## Abstract

**Background:**

Mice models suggest epigenetic inheritance induced by parental allergic disease activity. However, we know little of how parental disease activity before conception influences offspring's asthma and allergy in humans.

**Objective:**

We aimed to assess the associations of parental asthma severity, bronchial hyperresponsiveness (BHR), and total and specific IgEs, measured before conception vs. after birth, with offspring asthma and hayfever.

**Methods:**

The study included 4293 participants (mean age 34, 47% men) from the European Community Respiratory Health Survey (ECRHS) with information on asthma symptom severity, BHR, total and specific IgEs from 1991 to 1993, and data on 9100 offspring born 1972–2012. Adjusted relative risk ratios (aRRR) for associations of parental clinical outcome with offspring allergic disease were estimated with multinomial logistic regressions.

**Results:**

Offspring asthma with hayfever was more strongly associated with parental BHR and specific IgE measured *before* conception than *after* birth [BHR: aRRR = 2.96 (95% CI: 1.92, 4.57) and 1.40 (1.03, 1.91), respectively; specific IgEs: 3.08 (2.13, 4.45) and 1.83 (1.45, 2.31), respectively]. This was confirmed in a sensitivity analysis of a subgroup of offspring aged 11–22 years with information on parental disease activity both before and after birth.

**Conclusion & Clinical Relevance:**

Parental BHR and specific IgE were associated with offspring asthma and hayfever, with the strongest associations observed with clinical assessment *before* conception as compared to *after* birth of the child. If the hypothesis is confirmed in other studies, parental disease activity assessed before conception may prove useful for identifying children at risk for developing asthma with hayfever.

## Introduction

Familial aggregation of asthma and allergies has been known for many decades, with the first reports indicating a 40–60% risk of allergy if both parents were allergic 1. Later studies found that the immune system response towards environmental exposures differed by genotype 2, by gene‐by‐environment interactions 3. Exposures during early life, including the intra‐uterine period, are believed to be particularly important for the development of asthma and allergy due to the plasticity of the developing lungs and immune system 4, 5, 6. *In utero* exposures such as maternal dietary factors 7 or smoking 8 can modify risk of allergic airway disease possibly through epigenetic mechanisms 7, 9, 10.

New research from animal models suggests that immunological markers of allergic disease may induce epigenetic changes traceable in offspring 11, 12, 13. Epigenetic mechanisms appear to be involved in transmission of immunological profiles from parents to offspring 14, and immunological activity may modify epigenetic characteristics, as has been reported for airway inflammation in animal models 15, 16. Thus, asthma severity and clinical objective measures of parental respiratory or allergic disease activity, such as bronchial hyperresponsiveness (BHR) or serum IgE, might influence the risk of offspring disease, independent of genetic heritability. A recently published study, investigating age of onset of parental allergic disease, did not identify differences on risk of offspring asthma between parental asthma starting before or after birth of the child; results for rhinitis and eczema were less consistent 17. However, in one study, it was found that 50% of healthy parents of asthmatic children had bronchial hyper‐reactivity, suggesting that bronchial reactivity has an autosomal dominant pattern of inheritance 16. Therefore, to address hypotheses from animal models on whether preconception disease activity may influence offspring phenotype, objectively measured markers of parental disease activity before conception would be important, but such data are not available in most human studies.

Furthermore, there is evidence that parental exposure *before* conception might influence disease risk in offspring differentially through the maternal and paternal lines 18. Certain genetic alleles may be expressed to a greater level in male or female individuals, thereby contributing to sex‐specific heritability patterns 18, 19. A meta‐analyses of 33 studies concluded that a maternal history of asthma had greater impact on subsequent development of asthma in offspring than paternal asthma 20, while other studies report that paternal asthma or atopy infers the strongest risk for offspring asthma and allergy 17, 21, 22.

In this study, we aimed to test whether parental asthma severity, BHR, specific IgEs, and total IgE were associated with offspring risk of asthma and hayfever, and in particular whether the associations between parental and offspring characteristics differed if the parental markers were measured *before* conception as compared to *after* the child's birth. Further, we studied whether associations with offspring disease risk differed through the maternal and paternal line.

## Materials and methods

### Study population

The European Community Respiratory Health Survey (ECRHS) is a random population‐based multi‐centre cohort study of participants aged 20–44 years at the time of recruitment (1991–1993) 23. It included an initial screening questionnaire, and for a random subsample, extensive interviewer‐led questionnaires and clinical examinations with spirometry, methacholine challenge test, and blood sampling for measurements of total IgE and specific IgE towards common inhalant allergens. The clinical sample was enriched with participants who reported currently taking asthma medication or asthma attacks or shortness of breath at night during the last year. The first follow‐up (ECRHS II) took place in 2000–2002, and the second follow‐up (ECRHS III) was completed in 2013. Exposure assessment was based on parental disease status and clinical outcomes from ECRHS I, and the outcomes were defined by data on offspring asthma and hayfever collected in ECRHS III. Parental disease status (BHR and specific IgEs) assessed in ECRHS II was used to define post‐conception parental disease markers in a sensitivity analysis limited to a subgroup of offspring born between ECRHS I and ECRHS II. Persons who participated in ECRHS I and III who had at least one offspring born between 1972 and 2012 (twenty years before or after ECRHS I) were considered. Offspring born the year before or after the ECRHS I were excluded from analyses of parental disease activity before conception and after birth. Each centre obtained ethical approval from the appropriate institutional or regional ethics committee, and written consent was obtained from each participant.

### Parents’ clinical phenotypes characteristics from ECRHS I

#### Methacholine challenge test

Methacholine bronchial challenge tests were performed in all participants with no medical contraindication, unless baseline FEV_1_ was < 80% of predicted or FEV_1_ post‐saline dilution was lower than 90% of the baseline FEV_1_ 24. BHR was defined as a 20% reduction in FEV_1_ from the highest FEV_1_ post‐diluent (PD_20_) during the test with an accumulated dose of methacholine of 1 mg (PD_20_ ≤ 1 mg).

#### Asthma symptom score

Asthmatic symptoms during the previous 12 months, as reported in a standardized face‐to‐face interview, were summarized by a 0–5 range score based on the number of symptoms (wheeze and breathlessness, woken with chest tightness, woken by attack of shortness of breath, attack of shortness of breath at rest, attack of shortness of breath after exercise) 25.

#### Total and specific IgE

Total IgE and specific IgE to house dust mite, cat, timothy grass, and *Cladosporium herbarum* (mould) in serum samples were measured in a central laboratory by the CAP system (Thermo Fisher, Uppsala, Sweden), as described in detail elsewhere 26. Positive specific IgE was defined by IgE ≥ 0.35 kU/L to at least one of the four allergens.

### Asthma and hayfever in offspring

In ECRHS III, through a standardized face‐to‐face interview, participants who reported to have children were asked to report year of birth and the presence of asthma and hayfever for each child. Offspring asthma was defined as a positive answer to ‘has he/she ever had asthma before 10 years of age?’ or to ‘has he/she ever had asthma after 10 years of age?’ and hayfever by a positive response to ‘has he/she ever had hayfever/rhinitis?’ These questions defined the outcomes asthma only, hayfever only, both asthma and hayfever (asthma with hayfever) or none of these. ‘Asthma’ and ‘asthma with hayfever’ combined confirmative answer to asthma at either or both time‐points (before or after 10 years of age), and controls were those offspring who did not have hayfever or asthma neither before nor after 10 years of age.

### Statistical analysis

Associations between parental asthma symptom score, BHR, specific IgEs and total IgE measured in ECRHS I, and the four‐level variable offspring asthma alone, hayfever alone and asthma with hayfever reported in ECRHS III were estimated with multinomial logistic regressions. Relative risk ratio (RRR) estimates were obtained with GEE multinomial regression models to account for clustering effects within the families. To assess the importance of parental disease activity before conception as compared to after birth, models were stratified by parental disease activity measured before and after the child's birth. RRRs were reported for (i) one unit increase in asthma symptom score; (ii) positive BHR test; (iii) any specific IgE; and (iv) per log10 unit increase in total IgE. All models were adjusted for centre, type of samples (random or symptomatic), offspring age, sex and parity, and parental age, sex, smoking status (never, ex, or current smoker), and pack‐years. Paternal and maternal asthma symptom score, BHR, specific IgEs and total IgE, and offspring risk of disease were assessed in separate models on paternal and maternal disease activity and clinical markers. Because we have information available for only one of the child's parents, we did not include interaction terms in the models.

### Sensitivity analyses

In a subsample of participants having one child born before and one child born after the ECRHS I clinical examination, we assessed whether disease activity relative to when the child was born was important for offspring disease risk. Further sensitivity analyses were performed for offspring born 10 or 5 years before or after ECRHS I (to narrow the age difference between offspring born before and after ECRHS) and for offspring for which parental clinical assessment were performed both before and after birth (offspring born between ECRHS I and ECRHS II).

All statistical analyses were performed using R version 3.1 (R Core Team, 2016).

## Results

### Study population and characteristics

**Figure 1:**
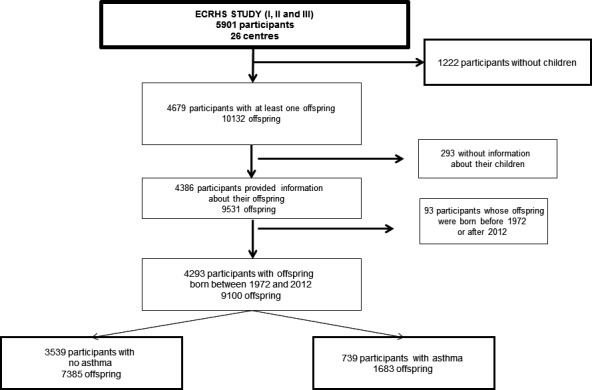
Flow chart of participants from ECRHS and their offspring included in this study.

A total of 5901 subjects participated in the clinical phase of ECHRS I and answered the ECRHS III questionnaire. After excluding participants with no children (*n* = 1222), missing information about the children (*n* = 293), and offspring born before 1972 or after 2012 (*n* = 93), the population in this study included 4293 participants and their offspring (*n* = 9100) (Fig. 1). Characteristics of the participants and their offspring included in the analyses are reported in Table 1. Overall, 63% of the offspring had been born at least one year before the ECRHS I, 6% were born in the year before or after ECRHS I, and 31% were born at least one year after ECRHS I (Table 1). At ECRHS III when offspring data were reported, the mean age of offspring born after ECRHS I was 12.9 years (SD = 4.5 years) and they had more siblings (two in average) than children born before ECRHS I (mean age 30 years (SD = 5.4) and 1.7 siblings on average), *P* < 0.001. Offspring born after ECRHS I had a higher prevalence of early‐onset asthma (14.5% vs. 9.9%, *P* < 0.001). Because they were younger with fewer contributing years, they had lower prevalence of late‐onset asthma (8.5% vs. 9.9%, *P* = 0.02) and hayfever (19.7% vs. 25.5%, *P* < 0.001) than children born before ECRHS I. The mothers reported 13% offspring asthma (early onset) and 28% hayfever compared to 9% and 20%, respectively, reported by the fathers (Table S1).

**Table 1:** Characteristics of index participants at ECRHS I and their offspring

	*N*	%
Characteristics of the offspring (*N* = 9100)		
Status		
Offspring born after ECRHS I clinical examination	2785	30.68
In uterus/early childhood	586	6.46
Offspring born before ECRHS I clinical examination	5706	62.86
Sex, men	4585	50.66
Age, year, mean (SD)	9100	24.1(9.3)
Asthma only	608	6.94
Hayfever only	1464	16.70
Asthma with hayfever	650	7.42
Parental characteristics at ECRHS I (*n* = 4293)		
Sex, men	2011	46.8
Age, year, mean (SD) at ECRHS I	4293	34.4(7)
From random sample	3670	85.5
Smoking status at ECRHS I		
Never smokers	1922	44.8
Ex‐smokers	973	22.7
Current smokers	1397	32.5
N of children	4293	1.1(0.3)
Asthma ever	739	17.27
Bronchial responsiveness, (*n* = 3467)		
Test not performed and FEV_1_% predicted ≤ 70%	46	1.3
Bronchial hyperresponsiveness+	495	14.3
Total IgE, μl, geom. mean (SD)	3770	30.7(4.80)

### Parental asthma severity, BHR, and IgEs before conception and after birth

**Figure 2:**
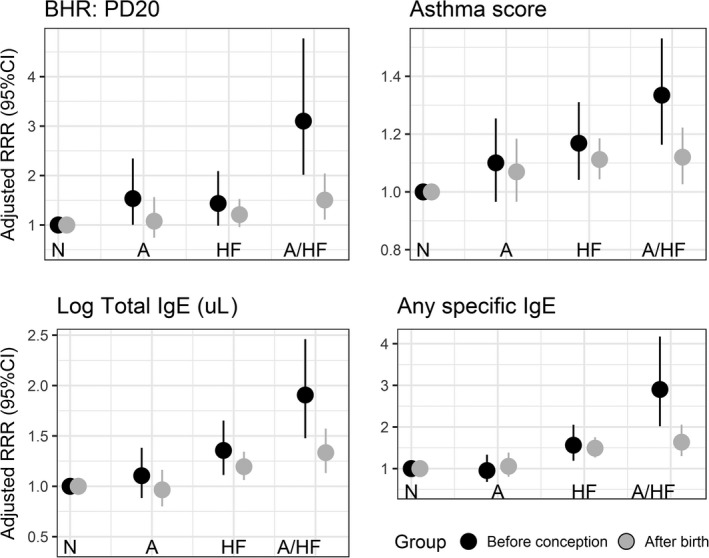
Parental disease activity markers in ECRHS I and asthma/hayfever in offspring stratified by clinical markers assessed before conception and after birth: N=No asthma/hayfever; A=Asthma; HF=Hayfever; A/HF: Asthma with hayfever.

**Figure 3:**
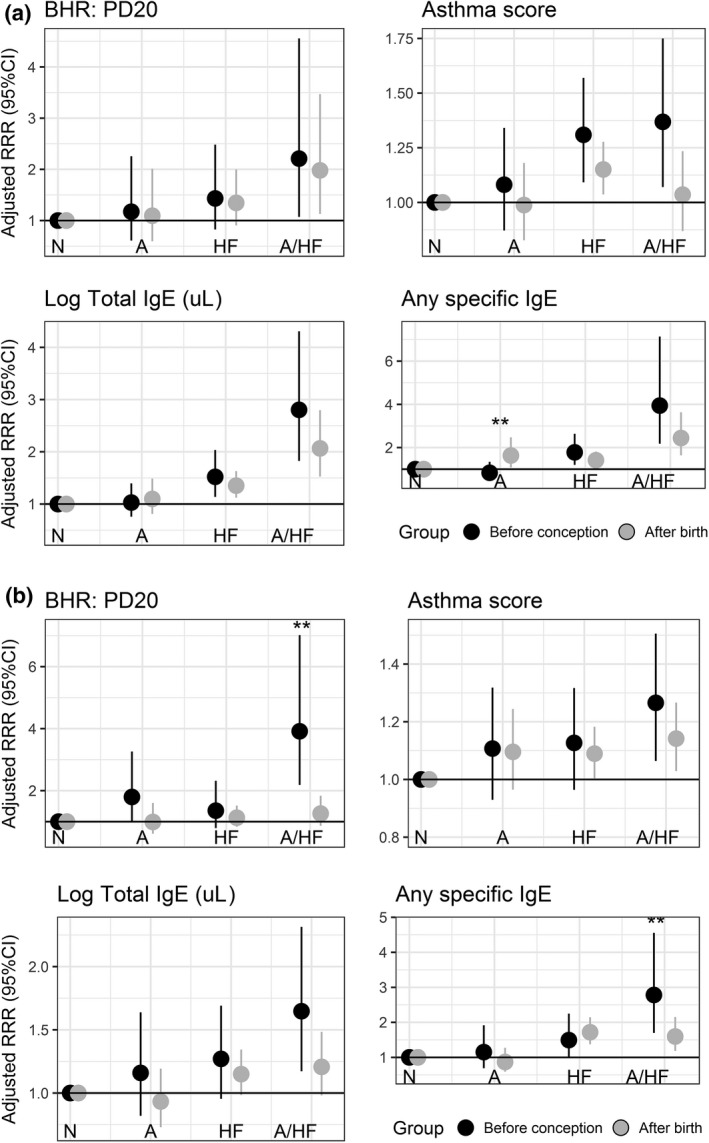
(a) Paternal disease activity markers in ECRHS I and asthma/hayfever in offspring stratified by clinical markers assessed before conception and after birth. (b) Maternal disease activity markers in ECRHS I and asthma/hayfever in offspring stratified by clinical markers assessed before conception and after birth: N=No asthma/hayfever; A=Asthma; HF=Hayfever; A/HF: Asthma with hayfever.

A positive BHR test before the child's birth was associated with a higher risk of asthma with hayfever [Adjusted relative risk ratios (aRRR) = 2.96 (1.92, 4.57)] compared to parental BHR measured post‐natally [aRRR = 1.40 (1.03, 1.92) (Table 2)]. A similar difference was observed for any positive specific IgE measured before conception compared to after birth of the child [aRRR = 3.08 (2.13, 4.45) and aRRR = 1.83 (1.45, 2.31), respectively (Table 2)], as well as for increasing asthma score and total IgE (Table 2 and Fig. 2). When similar analyses were performed for fathers and mothers separately, the risk of offspring asthma with hayfever was higher for increasing asthma score in the fathers before conception compared to after birth (Fig. 3a). Mothers’ BHR, any specific IgE, and higher total IgE before conception were stronger risk factors for offspring asthma with hayfever than the same maternal disease markers assessed after birth (Fig. 3b). To reduce the large age difference between the two offspring cohorts, we applied sensitivity analyses using more narrow time windows with offspring born 10 or 5 years before or after ECRHS I. For both models, the point estimates for offspring asthma with hayfever remained higher for parental disease activity (BHR and specific IgE) measured before rather than after conception (Table 3A and B). To eliminate potential cohort effects, we also compared one group of offspring for which parental clinical assessment was performed both before and after birth of the child (offspring born between ECRHS I and ECRHS II). The association between parental positive specific IgE measured before conception (ECRHS I) was more strongly associated with offspring asthma with hayfever than for parental positive specific IgE measured after conception (ECRHS II) (Table 3C). We performed a sensitivity analyses with a subgroup of participants who had one child born before and one child born after ECRHS I. Similar to the results reported above, the risk for offspring asthma with hayfever was higher for asthma symptom score, BHR, and specific and total IgEs assessed before rather than after conception (Table S2). We did further sensitivity analyses by including time elapsed between birth and the clinical assessments (ECRHS I) of the parents. There was no change in the estimates compared to the models without these adjustments (results not shown).

**Table 2:** Parental asthma score, bronchial hyperresponsiveness, specific and total IgE in ECRHS I and asthma and hayfever in offspring stratified by assessed before conception and after birth

Parental disease markers		Parental disease markers before conception (*N* = 1564)		Parental disease markers after birth (*N* = 2987)	
		Adjusted RRR (95% CI)	*P* value	Adjusted RRR^a^ (95% CI)	*P* value
		No offspring asthma/hayfever (ref)			
Offspring	Asthma score, per 1 unit increase in score (*N* = 4107)				
	Asthma only	1.10 (0.96, 1.25)	0.16	1.06 (0.96, 1.18)	0.24
	Hayfever only	1.16 (1.03, 1.30)	**0.001**	1.10 (1.04, 1.18)	**0.002**
	Asthma with hayfever	1.31 (1.15, 1.51)	**< 0.0001**	1.11 (1.02, 1.21)	**0.02**
Offspring	Bronchial responsiveness: PD20, yes vs. no (*N* = 3338)				
	Asthma only	1.53 (1.00, 2.35)	**0.05**	1.05 (0.73, 1.53)	0.79
	Hayfever only	1.40 (0.96, 2.04)	0.08	1.15 (0.91, 1.46)	0.24
	Asthma with hayfever	2.96 (1.92, 4.57)	**< 0.0001**	1.40 (1.03, 1.91)	**0.03**
Offspring	Any specific IgE, yes vs. no (*N* = 3629)				
	Asthma only	0.95 (0.68, 1.33)	0.76	1.13 (0.86, 1.49)	0.37
	Hayfever only	1.59 (1.21, 2.09)	**0.001**	1.57 (1.33, 1.85)	**< 0.001**
	Asthma with hayfever	3.08 (2.13, 4.45)	**< 0.001**	1.83 (1.45, 2.31)	**< 0.001**
Offspring	Total IgE, per log10 (IgE) unit (*N* = 3630)				
	Asthma only	1.10 (0.88, 1.38)	0.39	1.01 (0.83, 1.22)	0.94
	Hayfever only	1.38 (1.13, 1.68)	**0.002**	1.24 (1.10, 1.39)	**< 0.001**
	Asthma with hayfever	2.00 (1.54, 2.59)	**< 0.001**	1.43 (1.21, 1.69)	**< 0.001**

a Estimates were obtained with GEE multinomial regression models, adjusted for centre, sample, offspring age and sex, parental age and sex, parity, parental smoking status, and pack‐year.

**Table 3:** Parental bronchial hyperresponsiveness (BHR) and specific IgE, measured before conception and after birth of offspring, as associated with asthma and hayfever in offspring; (A) offspring born in a 10‐year window before and after parental measurement (in ECRHS I); (B) offspring born in a 5‐year window before and after parental measurement (in ECRHS I); and (C) offspring born between parental measurements in ECRHS I (preconception) and ECRHS II (after birth)

	Offspring asthma and hayfever *N* (%)	Parental disease markers assessed before conception		Parental disease markers assessed after birth	
		Adjusted RRR (95% CI)^a^	*P* value	Adjusted RRR (95% CI)^a^	*P* value
(A) Offspring born 10 years before or after ECRHS I (*N* = 3111)		*N* = 1411		*N* = 2081	
BHR (*N* = 2485): PD20, yes vs. no	323 (7.9)	3.28 (2.05, 5.23)	<0.001	1.61 (1.08, 2.40)	0.020
Any specific IgE (*N* = 2736)	371 (8.2)	3.18 (2.14, 4.72)	<0.001	1.87 (1.38, 2.52)	<0.001
(B) Offspring born 5 years before or after ECRHS I (*N* = 1964)		*N* = 964		*N* = 1242	
BHR (*N* = 1550): PD20, yes vs. no	168 (8.0)	3.35 (1.72, 6.53)	<0.001	1.48 (0.83, 2.65)	0.190
Any specific IgE (*N* = 1722)	193 (8.3)	2.61 (1.54, 4.44)	<0.001	2.12 (1.38, 3.26)	0.001
(C) Offspring born between ECRHS I and ECRHS II (*N* = 1530)		*N* = 1319		*N* = 1430	
BHR †(*N* = 1050 in EC I; *N* = 983 in EC II): PD20, yes vs. no	104 (7.1)/103 (7.3)	3.02 (1.81, 5.05)	<0.001	2.69 (1.58, 4.59)	<0.001
Any specific IgE †(*N* = 1138 in EC I; *N* = 1175 in EC II)	123 (7.8)/125 (7.4)	3.02 (2.00, 4.54)	<0.001	1.73 (1.15, 2.59)	0.008

a Estimates were obtained with multinomial regression models adjusted for centre, type of sample, offspring age, sex and parity, and parental age, sex, smoking status and pack‐year.

^†^Parental BHR and specific IgE as determined in ECRHS I were considered for the ‘before conception’ group and parental BHR and specific IgE as determined in ECRHS II were considered for the ‘after birth’ group.

### Paternal and maternal asthma severity, BHR and IgEs, and offspring risk of hayfever and asthma

Without taking timing of parental clinical assessment into account, increase in maternal and paternal asthma symptom score was associated with increased risk of offspring hayfever, and with asthma with hayfever, but the latter for mothers only (Table 4). Parental BHR, any specific IgE, and total IgE were associated with increased risk of offspring hayfever (for BHR only for fathers) and asthma with hayfever, with slightly stronger associations for paternal compared to maternal specific and total IgE (Table 4). When the analyses were stratified by gender of both the parent and the offspring, increasing asthma score among the parents was associated with an increased risk of offspring asthma with hayfever, but in sons only (Table S3). We observed stronger associations between paternal total IgE and asthma with hayfever in both sons and daughters (RRR = 2.31 (95% CI 1.72, 3.12) and 1.99 (95% CI 1.40, 2.85), respectively), as compared to maternal total IgE and asthma with hayfever in her sons and daughters (RRR = 1.31 (95% CI 1.04, 1.66) and 1.37 (95% CI 1.07, 1.77), respectively). A similar trend was seen for parental positive specific IgE and offspring asthma with hayfever (Table S3).

**Table 4:** Parental asthma score, bronchial hyperresponsiveness, specific and total IgE in ECRHS I and asthma and hayfever in offspring stratified by parents’ gender

Parental disease markers		Paternal (*N* = 1949)		Maternal (*N* = 2246)	
		Adjusted RRR (95% CI)^a^	*P* value	Adjusted RRR (95% CI)^a^	*P* value
		No offspring asthma/hayfever (ref)			
Offspring	Asthma score (*N* = 4171), per 1 unit increase in score				
	Asthma only	1.00 (0.88, 1.13)	0.96	1.10 (0.99, 1.21)	0.07
	Hayfever only	1.15 (1.06, 1.25)	**0.001**	1.08 (1.00, 1.15)	**0.04**
	Asthma with hayfever	1.12 (0.98, 1.27)	0.10	1.17 (1.07, 1.27)	**< 0.001**
Offspring	Bronchial responsiveness (*N* = 3381): PD20, yes vs. no				
	Asthma only	0.96 (0.63, 1.49)	0.87	1.31 (0.92, 1.87)	0.14
	Hayfever only	1.37 (1.02, 1.84)	**0.04**	1.17 (0.91, 1.50)	0.22
	Asthma with hayfever	1.88 (1.24, 2.86)	**0.003**	1.78 (1.32, 2.39)	**< 0.001**
Offspring	Any specific IgE (*N* = 3687)				
	Asthma only	1.15 (0.86, 1.55)	0.34	0.97 (0.73, 1.31)	0.86
	Hayfever only	1.48 (1.22, 1.81)	**< 0.001**	1.64 (1.36, 1.97)	**< 0.001**
	Asthma with hayfever	2.71 (1.99, 3.68)	**< 0.001**	1.82 (1.43, 2.31)	**< 0.001**
Offspring	Total IgE (*N* = 3688) per log10 (IgE) unit				
	Asthma only	1.11 (0.91, 1.36)	0.31	1.04 (0.86, 1.25)	0.71
	Hayfever only	1.38 (1.20, 1.60)	**< 0.001**	1.18 (1.04, 1.34)	**0.01**
	Asthma with hayfever	2.15 (1.72, 2.70)	**< 0.001**	1.35 (1.14, 1.60)	**0.001**

a Estimates were obtained with multinomial regression models, adjusted for offspring age and sex, parity, centre, sample, parental age, parental smoking status and pack‐years.

## Discussion

This is the first human study to investigate the link between parental respiratory and allergic disease activity measured before and after conception, and asthma and hayfever in offspring. We found that parental BHR and specific IgE positivity measured *before conception* was more strongly associated with offspring asthma and hayfever than disease activity measured *after birth*. This was most convincingly shown for mothers’ BHR and mothers’ specific IgE. When timing of clinical assessment relative to offspring birth was not taken into account, paternal clinical markers of allergic disease appeared to be generally more important than the mother's clinical markers for offspring allergic disease.

Shared environment appeared to be more important than genetic inheritance in a recent twin study 27. Effects of shared environment would result in stronger associations between parental and offspring disease for parental disease measured *after* birth of a child than before. On the other hand, we would expect the association between offspring and parental disease to be similar if this was only due to genetic heritability. However, we observed a stronger impact of parental BHR and IgEs measured *before* conception, suggesting that parental preconception disease activity in itself might affect respiratory health in offspring. Based on newly observed epigenetic inheritance in animal models 10, 11, 12, 13, 15 and our findings, we hypothesize that ongoing respiratory and allergic disease activity in humans might cause epigenetic changes that can be inherited to the next generation, manifesting phenotypic characteristics in the offspring. Although this is biologically plausible given experimental research results 11, 15, this has not been described in humans to date.

We assessed specifically asthma severity and clinical markers rather than parental history of allergic disease, and thus, our findings are not directly comparable with the analysis by Fuertes et al. 17 which focused on age of onset of parental allergic disease. They identified an association of maternal allergic rhinitis with offspring rhinitis only when maternal disease started before birth of the child 17. This is in agreement with our results on maternal specific and total IgE assessed before birth of the child being more strongly associated with offspring asthma with hayfever than when assessed after birth. It is known that asthma may worsen during pregnancy 28, but given that we included disease activity markers assessed before conception, this is unlikely to affect our results. Furthermore, becoming a parent might also possibly affect indicators of asthma and allergy. However, our sensitivity analyses of those offspring, where parental clinical markers were assessed both before and after birth of the child, supported the stronger association for clinical markers assessed before than after birth of the child.

Parental BHR was associated with an increased risk of asthma with hayfever in the offspring, and, to the best of our knowledge, this is the first study in humans to assess parental BHR in association with offspring risk of disease and with the possibility to differentiate between BHR measured before and after birth of the child. BHR varies with asthma activity, and although it is not specific for asthma, it reflects an important pathophysiological feature of asthma and is used in diagnosing asthma 29, 30 and in evaluating response to asthma treatment. We included a score of asthma symptoms as another indicator of asthmatic disease activity. This score is well described 25 and used in several studies. The findings from Fuertes et al. 17 that offspring asthma did not differ noticeably whether parental asthma started before or after the child's birth are in agreement with our findings that offspring asthma (without hayfever) was not differently associated with parental asthma score assessed before as compared to after birth of the child. Another study found that both maternal and paternal asthma symptoms were more common in parents of non‐atopic asthmatic children and that maternal BHR was associated with having a child with non‐atopic asthma 31. Objectively measured BHR should be a better measure of asthmatic disease activity than reported asthma symptoms or age of asthma debut. BHR has also been reported to be a common feature among healthy parents of asthmatic children 16 and thus indicates an link between this objective parental clinical markers and offspring’ asthma which would not have been found if only parental history of asthma or parental asthma symptoms had been assessed.

When considering both parental and offspring gender without taking timing of clinical assessment into account, paternal and maternal increasing asthma symptom score was associated with risk of asthma with hayfever in sons, but not in daughters. Previous publications have found maternal asthma to have a greater impact than paternal asthma on offspring asthma risk 20. However, in our study a clearer picture was observed for the association of paternal than for maternal clinical markers of allergic disease with offspring atopic disease when *not* taking into account whether the clinical markers were assessed before or after birth of the child. In the Isle of Wight Birth Cohort, Arshad and co‐authors reported that paternal but not maternal rhinitis was significantly associated with offspring rhinitis 21. Another recent paper found that post‐birth paternal (but not maternal) rhinitis was associated with higher prevalence of rhinitis in boys 17. However, when investigating parental disease activity before conception, we found more convincing impact of maternal disease activity. This is in agreement with a study by Cookson et al. 32 that reported stronger maternal than paternal inheritance of atopy.

In some subjects, immunological and clinical markers of allergic and respiratory disease were measured years before conception and we found that these markers predicted disease in offspring. In other subjects, immunological and clinical markers were measured after the offspring in question was born, and we found an association as expected – but the estimates appeared to be weaker than the associations with preconception measures. Sensitivity analysis of a subgroup of participants who had one child born before and one child born after the clinical assessment confirmed these findings and showed stronger associations of parental immunological and clinical markers measured before conception with offspring asthma and hayfever, than when these were measured after birth of the child. This was most consistently shown for parental bronchial hyperresponsiveness and for parental specific IgE positivity. Our analyses included offspring who were born 20 years before or after ECRHS I. Analyses limited to offspring aged 11–22 years of age reduced the potential for confounding by cohort effects and showed results consistent with analyses of total cohort. The findings were confirmed in sensitivity analyses which took into account more narrow time windows (cohorts of offspring born 10 and 5 years before and after the ECRHS I parental clinical examination).

The main strength of our study was the availability of detailed clinical information and well‐characterized phenotypes in a large sample of both mothers and fathers, which is often missing in birth cohort studies. To the best of our knowledge, this is the first study aimed at evaluating the association between parental objective (immunological and clinical) markers of disease with offspring asthma. The availability of longitudinal data allowed for analyses stratified according to whether offspring were born before or after the clinical assessment of the parents, and the large study population made it possible to study this in a subgroup of participants who contributed to offspring in both time‐points. The lack of information about offspring date of birth prevented us from analysing the *in utero* exposure to maternal disease activity.

Previous studies have shown that parents (in particular fathers) over‐reported their own atopic history if their child had an atopic illness 33. By applying clinical objective markers of parental allergic and respiratory diseases, we have avoided such bias in reporting. However, the information on offspring outcomes is based on questionnaire data and therefore subject to reporting bias; for example, the mothers reported higher prevalence of offspring outcomes than the fathers did. In a manuscript in preparation and abstract at the ERS congress 2016 34, we have shown that the agreement between parent and offspring report of offspring asthma was good for early‐onset asthma (kappa 0.70) and moderate for late‐onset asthma (kappa 0.47). Mothers were more likely to report offspring asthma correctly than did fathers; this is related in particular to mild cases. This may contribute to the higher prevalence of offspring outcomes reported by the mothers compared to the fathers in the present study. Nevertheless, our results show crudely similar associations between maternal and paternal disease markers (and slightly higher estimates for paternal allergic markers) and offspring disease in models not accounting for whether the offspring were born before or after the parental clinical assessment.

We cannot rule out the possibility that parents will better recall asthma and hayfever in offspring that are younger and likely to be living at home with the parents. However, hayfever is less prevalent in the youngest cohort, and some proportion of the offspring in the youngest cohort has not yet reached the age where hayfever will develop. Nevertheless, parental clinical phenotypes were most consistently associated with offspring asthma with hayfever in the youngest cohort, which may indicate that this is the most relevant phenotype in our study. Given that a higher prevalence of asthma with hayfever would be more likely to be present if the offspring had been older, it seems unlikely that this possibly underestimated prevalence of hayfever have led to stronger estimates for the youngest cohort as compared to the older offspring cohort, where the prevalence of hayfever is higher. Parent‐of‐origin effects on reporting of offspring phenotype could influence the sex‐specific patterns identified, but are unlikely to account for differences as related to objective markers of respiratory and allergic disease measured before and after conception.

In conclusion, our analyses suggest that parental disease activity (immunological and clinical markers of disease) measured *before conception* was more strongly related to offspring asthma and hayfever than parental disease activity measured *after birth*. We hypothesise that the observed patterns are more likely explained by epigenetic inheritance than by shared environment or genetics alone. This is an unexplored area and our paper mainly adds new hypotheses as to how parental disease activity might impact offspring disease. Our findings should be investigated further in epidemiologic and mechanistic studies, and if confirmed, it would be of major importance to investigate whether treatment or interventions in parents before conception would impact on disease risk in offspring.

## Sources of funding

This work is part of the Ageing Lungs in European Cohorts (ALEC) Study (www.alecstudy.org), which has received funding from the European Union's Horizon 2020 Research and Innovation Program under grant agreement No. 633212.

The following grants helped to fund the local studies:

Financial support for ECRHS I:

**Australia:** Asthma Foundation of Victoria, Allen and Hanbury's, **Belgium**: Belgian Science Policy Office, National Fund for Scientific Research, **Denmark:** The Danish Lung Association, **Estonia:** Estonian Science Foundation, grant no 1088, **France:** Ministère de la Santé, Glaxo France, Insitut Pneumologique d'Aquitaine, Contrat de Plan Etat‐Région Languedoc‐Rousillon, CNMATS, CNMRT (90MR/10, 91AF/6), Ministre delegué de la santé, RNSP, France; GSF, **Germany**: Bundesminister für Forschung und Technologie, **Greece:** The Greek Secretary General of Research and Technology, Fisons, Astra and Boehringer‐Ingelheim; **India:** Bombay Hospital Trust, **Italy:** Ministero dell'Università e della Ricerca Scientifica e Tecnologica, CNR, Regione Veneto grant RSF n. 381/05.93, **New Zealand:** Asthma Foundation of New Zealand, Lotteries Grant Board, Health Research Council of New Zealand, **Norway:** Norwegian Research Council project no. 101422/310; **Portugal:** Glaxo Farmacêutica Lda, Sandoz Portugesa, **Spain:** Fondo de Investigación Sanitaria (#91/0016‐060‐05/E, 92/0319 and #93/0393), Hospital General de Albacete, Hospital General Juan Ramón Jiménez, Dirección Regional de Salud Pública (Consejería de Sanidad del Principado de Asturias), CIRIT (1997 SGR 00079) and Servicio Andaluz de Salud; **Sweden:** The Swedish Medical Research Council, the Swedish Heart Lung Foundation, the Swedish Association against Asthma and Allergy; **Switzerland:** Swiss National Science Foundation grant 4026‐28099; **UK:** National Asthma Campaign, British Lung Foundation, Department of Health, South Thames Regional Health Authority**, USA:** United States Department of Health, Education and Welfare Public Health Service (grant #2 S07 RR05521‐28).

Financial Support for ECHRS III

**Australia**: National Health & Medical Research Council**, Belgium: Antwerp South, Antwerp City:** Research Foundation Flanders (FWO), grant code G.0.410.08.N.10 (both sites), **Denmark:** The Faculty of Health, Aarhus University No. 240008, The Wood Dust Foundation No. 444508795, **Estonia: Tartu‐** SF0180060s09 from the Estonian Ministry of Education. **France: (All)** Ministère de la Santé. Programme Hospitalier de Recherche Clinique (PHRC) national 2010 **Bordeaux:** INSERM U897 Université Bordeaux segalen **Grenoble:** Comitee Scientifique AGIRadom 2011. **Paris:** Agence Nationale de la Santé, Région Ile de France, domaine d'intérêt majeur (DIM) **Germany : Erfurt:** German Research Foundation HE 3294/10‐1 **Hamburg**: German Research Foundation MA 711/6‐1, NO 262/7‐1 **Iceland:** Reykjavik, The Landspitali University Hospital Research Fund, University of Iceland Research Fund, ResMed Foundation, California, USA, Orkuveita Reykjavikur (Geothermal plant), Vegagerðin (The Icelandic Road Administration (ICERA)). **Italy:** All Italian centres were funded by the Italian Ministry of Health, Chiesi Farmaceutici SpA, and in addition, **Verona** was funded by Cariverona Foundation, Education Ministry (MIUR). **Norway:** Norwegian Research Council grant no 214123, Western Norway Regional Health Authorities grant no 911631, Bergen Medical Research Foundation. **Spain:** Fondo de Investigación Sanitaria (PS09/02457, PS09/00716 09/01511, PS09/02185, PS09/03190), Servicio Andaluz de Salud, Sociedad Española de Neumología y Cirurgía Torácica (SEPAR 1001/2010); **Barcelona:** Fondo de Investigación Sanitaria (FIS PS09/00716) **Galdakao:** Fondo de Investigación Sanitaria (FIS 09/01511) **Huelva**: Fondo de Investigación Sanitaria (FIS PS09/02185) and Servicio Andaluz de Salud **Oviedo:** Fondo de Investigación Sanitaria (FIS PS09/03190) **Sweden:** All centres were funded by The Swedish Heart and Lung Foundation, The Swedish Asthma and Allergy Association, The Swedish Association against Lung and Heart Disease. **Swedish Research Council for health, working life and welfare (FORTE) Göteborg :** Also received further funding from the Swedish Council for Working Life and Social Research. Umea also received funding from Vasterbotten Country Council ALF grant. **Switzerland:** The Swiss National Science Foundation (grants no 33CSCO‐134276/1, 33CSCO‐108796, 3247BO‐104283, 3247BO‐104288, 3247BO‐104284, 3247‐065896, 3100‐059302, 3200‐052720, 3200‐042532, 4026‐028099) The Federal Office for Forest, Environment and Landscape, The Federal Office of Public Health, The Federal Office of Roads and Transport, the Canton's Government of Aargan, Basel‐Stadt, Basel‐Land, Geneva, Luzern, Ticino, Valais and Zürich, the Swiss Lung League, the Canton's Lung League of Basel Stadt/Basel, Landschaft, Geneva, Ticino, Valais and Zurich, SUVA, Freiwillige Akademische Gesellschaft, UBS Wealth Foundation, Talecris Biotherapeutics GmbH, Abbott Diagnostics, European Commission 018996 (GABRIEL), Wellcome Trust WT 084703MA, **UK:** Medical Research Council (Grant Number 92091), Support also provided by the National Institute for Health Research through the Primary Care Research Network. The coordination of the ECRHS III was funded through the Medical Research Council (Grant Number 92091).

## Author contributions

RJB drafted the manuscript, MR contributed to the drafting of the manuscript and with statistical analysis, CS and SCD contributed to conception and design of the study, and all authors contributed to data collection for their study centre, interpretation of data for the work and revising the manuscript critically for important intellectual content. All authors approved the final version of the manuscript.

## Conflict of interest

The authors declare no conflict of interest.

## Supporting information


**Table S1.** Offspring phenotypes as reported by fathers and mothers.
**Table S2.** Association between asthma severity and clinical markers and offspring asthma and hay fever only for parents who have one child born before and one child born after ECRHS I.
**Table S3.** Association between parental asthma score, BHR, specific and total IgE and atopic status and asthma and allergy in children, stratified by gender of offspring and parents.
