# Supplementary material for: Clinical markers of asthma and IgE assessed in parents before conception predict asthma and hayfever in the offspring
Source: Clin Exp Allergy. 2017 Mar 28;47(5):627–38. doi: 10.1111/cea.12906 (PMC5447870; doi:10.1111/cea.12906)
Supplement: Supplementary file 1 — Table S1. Offspring phenotypes as reported by fathers and mothers. Table S2. Association between asthma severity and clinical markers and offspring asthma and hay fever only for parents who have one child born before and one child born after ECRHS I. Table S3. Association between parental asthma score, BHR, specific and total IgE and atopic status and asthma and allergy in children, stratified by gender of offspring and parents. [file CEA-47-627-s001.docx]

**Table S1**: Offspring phenotypes as reported by fathers and mothers

| **Offspring phenotypes** | | **Reporting parents’ gender** | | ***P* value** |
| --- | --- | --- | --- | --- |
|  |  | *Fathers*  *(N=2011 subjects, 4328 offspring)* | *Mothers*  *(N=2282 subjects, 4772 offspring)* |  |
| Never asthma^a^ |  | 3657 (90.5%) | 3866 (86.8%) | <0.001 |
| Early onset asthma (yes) | | 383 (9.48%) | 589 (13.2%) |  |
| Missing | | 288 (6.65%) | 317 (6.64%) |  |
| Never asthma^a^ |  | 3657 (91.8%) | 3866 (89.1%) | <0.001 |
| Late onset asthma(yes) | | 326 (8.18%) | 475 (10.9%) |  |
| Missing | | 345 (7.97%) | 431 (9.03%) |  |
| Hay fever | No | 3441 (79.5%) | 3446 (72.2%) | <0.001 |
|  | Yes | 848 (19.6%) | 1293 (27.1%) |  |
|  | Missing | 39 (0.90%) | 33 (0.69%) |  |
| Asthma only | | 269 (6.50%) | 339 (7.33%) | <0.001 |
| Hay fever only | | 610 (14.7%) | 854 (18.5%) |  |
| Asthma with hay fever | | 223 (5.39%) | 427 (9.23%) |  |
| No asthma/hay fever | | 3038 (73.4%) | 3004 (65.0%) |  |
| Missing | | 34 (0.79%) | 39 (0.82%) |  |

^a^No asthma neither before nor after 10 years of age

**Table S2.** Association between asthma severity and clinical markers and offspring asthma and hay fever only for parents who have one child born before and one child born after ECRHS I.

|  |  | Parental disease markers assessed before conception | | Parental disease markers assessed after birth | |  |
| --- | --- | --- | --- | --- | --- | --- |
|  | **Parental disease markers** | **Adjusted OR**  **(95%CI)^a^** | ***P* value** | **Adjusted OR**  **(95% CI) ^a^** | ***P* value** | ***P* for interactions** |
| *No offspring asthma/hay fever (ref)* | | **N=565** |  | **N=625** |  |  |
|  | **Asthma score** |  |  |  |  |  |
| *Offspring* | *Asthma only* | 0.98 (0.68,1.42) | 0.92 | 1.39 (1.01,1.91) | 0.05 | 0.61 |
|  | *Hay fever only* | 1.36 (1.05,1.77) | 0.02 | 1.34 (1.08,1.65) | 0.007 | 0.88 |
|  | *Asthma with hay fever* | 1.61 (1.16,2.24) | 0.005 | 0.94 (0.65,1.36) | 0.73 | 0.06 |
|  | **BHR (N=2953)** |  |  |  |  |  |
| *Offspring* | *Asthma only* | 1.27 (0.41,3.90) | 0.68 | 4.02 (1.33,12.2) | 0.01 | 0.61 |
|  | *Hay fever only* | 2.14 (0.88,5.17) | 0.09 | 1.35 (0.62,2.95) | 0.45 | 0.74 |
|  | *Asthma with hay fever* | 3.36 (1.08,10.5) | 0.04 | 0.88 (0.26,2.96) | 0.83 | 0.02 |
| *Offspring* | **Specific IgE** |  |  |  |  |  |
|  | *Asthma only* | 1.09 (0.46,2.58) | 0.84 | 1.35 (0.56,3.24) | 0.50 | 0.74 |
|  | *Hay fever only* | 1.08 (0.57,2.05) | 0.81 | 1.59 (0.96,2.65) | 0.07 | 0.64 |
|  | *Asthma with hay fever* | 2.73 (1.15,6.48) | 0.02 | 1.50 (0.66,3.44) | 0.34 | 0.09 |
|  | **Total IgE** |  |  |  |  |  |
| *Offspring* | *Asthma only* | 1.26 (0.73,2.18) | 0.41 | 1.21 (0.67,2.19) | 0.52 | 0.74 |
|  | *Hay fever only* | 0.96 (0.61,1.51) | 0.86 | 1.44 (0.98,2.10) | 0.06 | 0.28 |
|  | *Asthma with hay fever* | 1.96 (1.06,3.62) | 0.03 | 1.51 (0.82,2.79) | 0.19 | 0.39 |

^a^ Estimates were obtained with GEE multinomial regression models, adjusted for centre, type of sample, offspring age, sex and parity, and parental age and sex, smoking status and pack-years

**Table S3:** Association between parental asthma score, BHR, specific and total IgE and atopic status and asthma and allergy in children, stratified by gender of offspring and parents

|  | **Sons (N=3067)** | | | | **Daughters (2993)** | | | |
| --- | --- | --- | --- | --- | --- | --- | --- | --- |
|  | **Fathers (N=1458)** | | **Mothers (1609)** | | **Fathers (1378)** | | **Mothers (1606)** | |
|  | Adjusted RRR  (95 % CI) **^a^** | *P* value | Adjusted RRR  (95 % CI) **^a^** | *P* value | Adjusted RRR  (95 % CI) **^a^** | *P* value | Adjusted RRR  (95 % CI) **^a^** | *P* value |
| **Parental asthma score (N=3171), per unit increase in score** | | | | |  |  |  |  |
| *Offspring asthma only* | 0.98 (0.82,1.17) | 0.810 | 1.13 (0.98,1.29) | 0.080 | 1.02 (0.84,1.24) | 0.820 | 1.07 (0.92,1.23) | 0.390 |
| *Offspring hay fever only* | **1.25 (1.11,1.40)** | **< 0.001** | 1.09 (0.98,1.20) | 0.100 | 1.05 (0.92,1.20) | 0.430 | 1.07 (0.97,1.18) | 0.200 |
| *Offspring asthma with hay fever* | **1.15 (0.97,1.37)** | **0.010** | **1.22 (1.08,1.37)** | **0.001** | 1.09 (0.89,1.34) | 0.390 | 1.11 (0.98,1.26) | 0.110 |
| **Parental BHR: PD20, yes vs no (N=2679)** | |  |  |  |  |  |  |  |
| *Offspring Asthma only* | 0.91 (0.51,1.63) | 0.760 | **1.67 (1.03,2.71)** | **0.040** | 1.01 (0.51,1.98) | 0.980 | 1.00 (0.58,1.73) | 0.990 |
| *Offspring hay fever only* | **1.71 (1.15,2.54)** | **0.008** | 1.22 (0.87,1.71) | 0.250 | 1.07 (0.68,1.70) | 0.760 | 1.14 (0.78,1.67) | 0.490 |
| *Offspring Asthma with hay fever* | **1.81 (1.02,3.20)** | **0.040** | **2.00 (1.34,3.00)** | **<0.001** | **2.12 (1.12,4.01)** | **0.020** | **1.64 (1.03,2.60)** | **0.040** |
| **Parental any specific IgE (N=2852), yes vs no** | |  |  |  |  |  |  |  |
| *Offspring asthma only* | 1.01 (0.68,1.50) | 0.970 | 1.36 (0.92,2.00) | 0.120 | 1.39 (0.89,2.17) | 0.140 | **0.63 (0.40,1.00)** | **0.050** |
| *Offspring hay fever only* | **1.34 (1.02,1.76)** | **0.040** | **1.51 (1.17,1.95)** | **0.002** | **1.72 (1.29,2.31)** | **< 0.001** | **1.84 (1.40,2.41)** | **< 0.001** |
| *Offspring Asthma with hay fever* | **2.99 (1.99,4.49)** | **< 0.001** | **1.66 (1.19,2.32)** | **0.003** | **2.43 (1.51,3.91)** | **< 0.001** | **2.01 (1.42,2.86)** | **< 0.001** |
| **Parental total IgE (N=2853), per log10 (IgE) unit** | |  |  |  |  |  |  |  |
| *Offspring asthma only* | 0.91 (0.69,1.19) | 0.480 | 1.17 (0.90,1.52) | 0.250 | **1.46 (1.07,2.01)** | **0.020** | **0.89 (0.67,1.17)** | **0.400** |
| *Offspring hay fever only* | **1.27 (1.04,1.54)** | **0.020** | 1.06 (0.89,1.27) | 0.500 | **1.56 (1.26,1.94)** | **< 0.001** | **1.33 (1.10,1.62)** | **0.003** |
| *Offspring asthma with hay fever* | **2.31 (1.72,3.12)** | **< 0.001** | **1.31 (1.04,1.66)** | **0.020** | **1.99 (1.40,2.85)** | **< 0.001** | **1.37 (1.07,1.77)** | **0.010** |

^a^ Estimates were obtained with GEE multinomial regression models, adjusted for centre, type of sample, offspring age, sex and parity, and parental age, smoking status and pack-years
